# Acidic Metal-Based Functional Ionic Liquids Catalyze the Synthesis of Bio-Based PEF Polyester

**DOI:** 10.3390/polym16010103

**Published:** 2023-12-29

**Authors:** Qiao Zhou, Yuanyuan Zhao, Yafei Shi, Rongrong Zheng, Liying Guo

**Affiliations:** School of Petrochemical Engineering, Shenyang University of Technology, Liaoyang 111003, China; zq18809861931@163.com (Q.Z.); 19824918195@163.com (Y.Z.); 15140998063@163.com (Y.S.); zhengrongrong2006@126.com (R.Z.)

**Keywords:** ionic liquid, catalyst, biobased, direct esterification, poly(ethylene 2,5-furandicarboxylate)

## Abstract

Utilizing triethylenediamine (DA), 1,3-propanesultone (PS), whose ring opens during the formation of the dizwiterion-intermediate DA-2PS, and the metal chlorides XCly, where X = Sn(IV), Zn(II),Al(III), Fe(III) and Mn(II), are used for the synthesis of five kinds of acidic metal-based functionalized ionic liquid catalysts ([DA-2PS][XCly]_2_). Their chemical structures, thermal stability and dual acidic active site were analyzed. We investigated the performance of [DA-2PS][XCly]_2_ in catalyzing the esterification reaction between 2,5-furandicarboxylic acid (FDCA) and ethylene glycol (EG) to synthesize poly (ethylene 2,5-furandicarboxylate)(PEF). Among the catalysts tested, [DA-2PS][SnCl_5_]_2_ exhibited the best catalytic performance under identical process parameters, and the optimal catalyst dosage was determined to be 0.05 mol% based on FDCA. The optimal conditions for the reaction were predicted using response surface methodology: a feed ratio of EG:FDCA = 1.96:1, an esterification temperature of 219.86 °C, a polycondensation temperature of 240.04 °C and a polycondensation time of 6.3 h, with a intrinsic viscosity of 0.67 dL·g^−1^. The resulting PEF was experimentally verified to exhibit an intrinsic viscosity of 0.68 dL·g^−1^ and a number average molecular weight of 28,820 g·mol^−1^. Finally, the structure and thermal properties of PEF were characterized. The results confirmed that PEF possessed the correct structure, exhibited high thermal stability and demonstrated excellent thermal properties.

## 1. Introduction

With the development of society and scientific and technological progress, people’s material well-being is increasing, resulting in a significant rise in the production and variety of plastic products. One such product is polyethylene terephthalate (PET), a petroleum-based polyester that has significantly enhanced people’s lives across various industries. However, with the depletion of petroleum resources and the rapid increase in white pollution, it is urgently necessary to find environmentally friendly green polyester that can effectively replace petroleum-based PET.

Poly (ethylene 2,5-furandicarboxylate) (PEF) is a bio-based polyester derived from lignocellulose [1,2]. Its raw material, 2,5-furandicarboxylic acid (FDCA), has been recognized as one of the 12 most promising bio-based platform compounds since 2004 [3]. PEF is considered a viable alternative to PET by many researchers, mainly because of the similar structure between the ring-conjugated bis-hydroxyl groups of FDCA and terephthalic acid (PTA) [4,5]. Not only does PEF exhibit properties comparable to PET, such as thermal stability, compressive strength, mechanical strength, Young’s modulus, tensile strength and glass transition temperature, but it also possesses significantly superior gas barrier performance. Furthermore, PEF can be produced entirely through biotechnology and offers enhanced recyclability in key applications, leading to a reduction of over 50% in CO_2_ emissions compared to PET [6,7,8].

The main synthesis methods of PEF are ring-opening polymerization, solution polycondensation, enzyme-catalyzed polymerization, melt solid-phase polycondensation and melt polycondensation. The ring-opening polymerization method can effectively inhibit the product discoloration, shorten the reaction time and avoid producing by-products, but there are disadvantages such as complex reaction conditions, low reactant reaction rate and high cost with pollution [9,10,11]. Solution polycondensation allows for better control of the process and can effectively increase the molecular weight of the product, but the process is more difficult to operate and consumes more energy [12,13]. The enzyme-catalyzed method is not harmful to the environment, but the process is not efficiently catalyzed by enzymes and the molecular weight of the product is low [14]. Melt solid-phase polycondensation produces products with high molecular weights, but they are also costly and not suitable for industrial production [15,16]. The melt polycondensation ester exchange method requires low purity of reactants, but has a long and complicated reaction time [17]. The melt polycondensation direct esterification method presents the advantages of high efficiency, low energy consumption, short time, stable process and high molecular weight of the product in the whole process, which is a more mature method for producing polyester PEF [18,19,20].

The PEF synthesis process primarily utilizes metal catalysts and organic enzyme catalysts [21,22,23,24]. Metal catalysts exhibit superior catalytic effects in the PEF synthesis reaction, but they often result in products with dark color, poor stability and toxicity issues. In order to address these limitations, alternative catalysts can be employed in the PEF synthesis process. Recently, organic enzyme catalysts have been introduced as potential substitutes for metal catalysts; however, their adoption is limited due to the complex preparation process and low catalytic efficiency.

Ionic liquids are widely used in various fields such as electrochemistry, organic synthesis, catalysis and extraction due to their advantageous properties, including high structural designability, good thermal stability and low vapor pressure [25,26,27,28]. Ionic liquids can be categorized as acidic, neutral and basic according to the difference in acidity provided by anions and cations. In polyester synthesis, acidic ionic liquids are widely used due to their adjustable acidity and uniform distribution of acid strength [29,30,31,32]. Zhao et al. prepared three biacidic (Brønsted acid and Lewis acid) tin-based functional ionic liquid catalysts. They were then used to catalyze the coupling reaction between ethylene carbonate (EC) and dimethyl succinate (DMSu) to synthesize PES. Among them, the catalyst containing sulfonic groups with the strongest Brønsted acidity ([HO_3_S-(CH_2_)_3_-mim]Cl-SnCl_4_) produced the best catalytic effect. Under the optimal process conditions for the transesterification reaction (temperature: 227 °C; material ratio: EC:DMSu = 1; catalyst consumption: 1.0 wt%; time: 5.5 h), PES had a yield of 67.12% and a selectivity of 82.47% [33].

In this study, we developed an acidic metal-based functional ionic liquid catalyst that exhibits high stability and catalytic activity for the synthesis of PEF by direct esterification of FDCA with ethylene glycol (EG). We compared the performance of this catalyst with traditional catalysts and common metal-free ionic liquid catalysts to assess the feasibility of using ionic liquid-catalyzed synthesis for PEF. Additionally, we investigated the impact of introducing metal groups on the catalytic performance of ionic liquid catalysts. The findings of this study provide important theoretical foundations and data support for the industrial production of PEF through direct esterification.

## 2. Experimental Section

### 2.1. Main Experimental Reagents

FDCA (with a mass fraction of 99.5%), Jiaxing Ruiyuan Biotech Co., Ltd. (Jiaxing, China); 1,3-propanesultone, AR, Guangzhou Hongcheng Biotechnology Co., Ltd. (Guangzhou, China); HCl, with a mass fraction 36%; AR, Liaoning Xinxing Reagent Co., Ltd. (Tieling, China); tetrachloroethane, (CH_3_COO)_2_Mn, ZnCl_2_ and AlCl_3_, both AR, Tianjin Damao Chemical Reagent Factory (Tianjin, China); triethylene diamine, ethylene glycol, SnCl_4_·5H_2_O, FeCl_3_·6H_2_O, MnCl_2_·4H_2_O, 1,8-diazabicyclo [5.4.0]undec-7-ene(DBU), hexafluoroisopropanol, dimethyl sulfoxide-d and tetramethylsilane, both AR, Shanghai Macklin Biochemical Co., Ltd. (Shanghai, China); ethyl acetate, AR, Tianjin Beichen District Fangzheng Reagent Factory (Tianjin, China); phenol, AR, Shanghai Aladdin Bio-chemical Technology Co., Ltd. (Shanghai, China); distilled water, [HO_3_S-(CH_2_)_3_-mim]Cl and [HO_3_S-(CH_2_)_3_-mim]SnCl_5_ self-prepared in the laboratory; nitrogen (99.99% purity), PetroChina Liaoyang Petrochemical Company (Liaoyang, China).

### 2.2. Methods of Investigation

FT-IR (Fourier transform infrared spectroscopy) analysis of ionic liquid and PEF was performed using an FTIR spectrometer (MAGNA-IR750, Bruker Optics, Leipzig, Germany) instrument using KBr as the blank in the range of 4000–500 cm^−1^.

Nuclear Magnetic Resonance Spectroscopy (^1^H NMR) measurement was carried out on a BRUKER III HD 400-MHz NMR spectrometer. Ionic liquid and PEF was dissolved in dimethyl sulfoxide-d (DMSO) and trifluoroacetic acid-d (TFA), respectively, with tetramethylsilane (TMS) as the internal reference.

The TG analysis (Thermogravimetric Analysis) of ionic liquid and PEF was obtained using a TGA4000 analyzer (NETZSCH Scientific Instruments Trading Ltd., Selb, Germany) at a heating rate of 10 °C/min under nitrogen atmosphere from 25 to 600 °C.

X-ray diffraction (XRD) measurements of ionic liquid were conducted on a ULTIMA instrument using Cu Kα radiation at 40 kV and 40 mA, with a wide-angle scanning range 2θ of 10°–80° and a scanning speed of 5°/min.

Glass-transition temperature and melting behavior were recorded with differential scanning calorimetry on DSC822e thermal analyzer (Mettler Toledo, Knonau, Switzerland), calibrated with indium standard. PEF samples were heated to 250 °C at a heating rate of 10 °C/min and kept for 10 min in order to erase the thermal history. The melt was cooled to 25 °C and then heated to 250 °C at 10 °C/min.

Number-average molecular weight (Mn) of PEF was obtained by gel permeation chromatography using a liner hexafluoroisopropanol (HFIP) column and a Waters 515 HPLC with OPTILAB DSP interferometric refractometer (Wyatt Technology, Santa Barbara, CA, USA) as the detector. The eluent was HFIP at a flow rate of 1.0 mL/min at 40 °C. Mono-dispersed polymethyl methacrylate was used as the standard samples.

Intrinsic viscosity [η] of the PEF was measured at 25 °C with an Ubbelohde viscometer. The concentration of the polyesters in a mixed solvent, phenol/1,1,2,2-tetrachloroethane (1/1, *w*/*w*), was 0.5 dL·g^−1^.

The contents of FDCA in the esterification products were determined by external reference method of HPLC on 1290 high-performance liquid chromatograph at room temperature. A concentration of 1 mg/mL for the samples was prepared by dissolving them in DMSO and FDCA, and used as the reference sample. The determination of FDCA conversion was conducted using the method described in the literature [32].

### 2.3. Synthesis of Acidic Metal-Based Functional Ionic Liquid Catalysts

#### 2.3.1. Synthesis of Intermediate I [DA-2PS]

A total of 11.22 g (0.1 mol) of DA was weighed in a round-bottomed flask while 100 mL of ethyl acetate was added as a protective solvent, 24.43 g (0.2 mol) of PS was added slowly to the flask under magnetic stirring, and the reaction was stirred under nitrogen protection for 48 h at room temperature. At the end of the reaction, filtration was carried out under reduced pressure, and the filter cake was washed with ethyl acetate five times, evaporated, and dried under vacuum for 24 h. A white solid powder was obtained and recorded as intermediate I.

#### 2.3.2. Synthesis of Intermediate II [DA-2PS][Cl]_2_

Take the above 3.60 g (0.01 mol) of intermediate I in a three-necked flask and dissolve it with a small amount of distilled water, add 100 mL of 0.2 mol/L dilute hydrochloric acid solution slowly dropwise into the three-necked flask, stir magnetically at room temperature for 1 h, then warm up to 80 °C to continue the reaction with magnetic stirring, and condense and reflux under the protection of nitrogen gas for 8 h. At the end of the reaction, evaporate by spinning and dry in vacuum to obtain a white viscous liquid, recorded as intermediate II.

#### 2.3.3. Synthesis of Acidic Metal-Based Ionic Liquid Catalyst [DA-2PS][XCly]_2_

**Figure 1:**
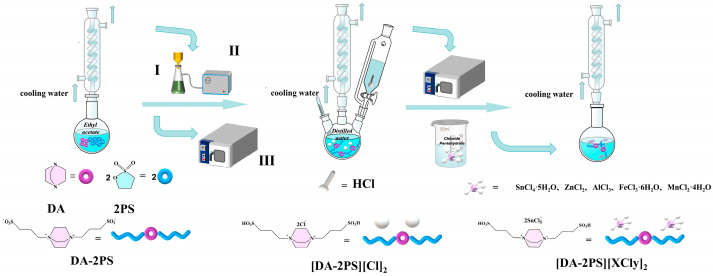
Diagram of the preparation of acidic metal-based functional ionic liquid catalysts (I is a Buchner funnel, II is a vacuum pump, III is a vacuum drying cabinet).

Using Lewis acid metal Sn as an example, take 4.31 g (0.01 mol) of intermediate II in a three-necked flask under an atmosphere of N_2_ and heat at 40 °C to make all of them melt. With magnetic stirring and rapid addition of 7.01 g (0.02 mol) SnCl_4_·5H_2_O, warm up to 90 °C and continue magnetic stirring, condensing, and refluxing the reaction for 6 h. After the reaction, the catalyst [DA-2PS][SnCl_5_]_2_ was obtained by spin evaporation and vacuum drying at 80 °C for 24 h. Other metal-based functional ionic liquid catalysts were prepared in the same way, and the preparation process is shown in Figure 1.

### 2.4. Investigation of the Performance of Acidic Metal-Based Functional Ionic Liquid Catalysts

**Figure 2:**
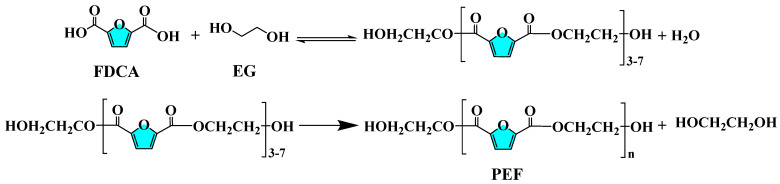
Reaction formula for the synthesis process of bio-based polyester PEF.

PEF was synthesized by catalyzing the reaction of FDCA with EG using direct esterification method with the five acidic metal-based functional ionic liquids prepared above as catalysts. The synthesis process is shown in Figure 2 in the following steps:

(1) Esterification reaction: 46.83 g (0.3 mol) of FDCA was weighed and 29.79 g (0.48 mol) of EG was mixed and added to a 250 mL four-necked flask fitted with a mechanical stirring and reduced pressure distillation apparatus, and 0.00045 mol of catalyst (0.15 mol% based on FDCA) was added rapidly under N_2_ atmosphere. Subsequently, mechanical stirring was turned on and the reaction system was heated to 60 °C, and nitrogen was continuously passed to replace the air in the device. Then, the temperature was slowly increased to 190 °C and the reaction continued at atmospheric pressure, during which the water generated was separated and collected in a timely manner. After reaching the clarification point, the reaction was continued until the temperature at the top of the column dropped below 80 °C, and the reaction was completed. The conversion of FDCA in this reaction was calculated according to [34].
(2) Condensation reaction: open the vacuum device, raise the temperature to 240 °C, vacuum decompression to −0.1 MPa and react for 5 h. As the process continues to collect the generated glycol, vacuum drying for 24 h is used to finally obtain the polymerization product PEF. The intrinsic viscosity (η) of PEF was determined according to the national standard GB/T 12005.1-1989 [35] with the methods reported in the literature [36].

## 3. Results and Discussion

### 3.1. Chemical Structure Characterization of Acidic Metal-Based Functional Ionic Liquid Catalysts

An FT-IR spectrometer was used for infrared characterization of the five acidic metal-based ionic liquid catalysts prepared.

**Figure 3:**
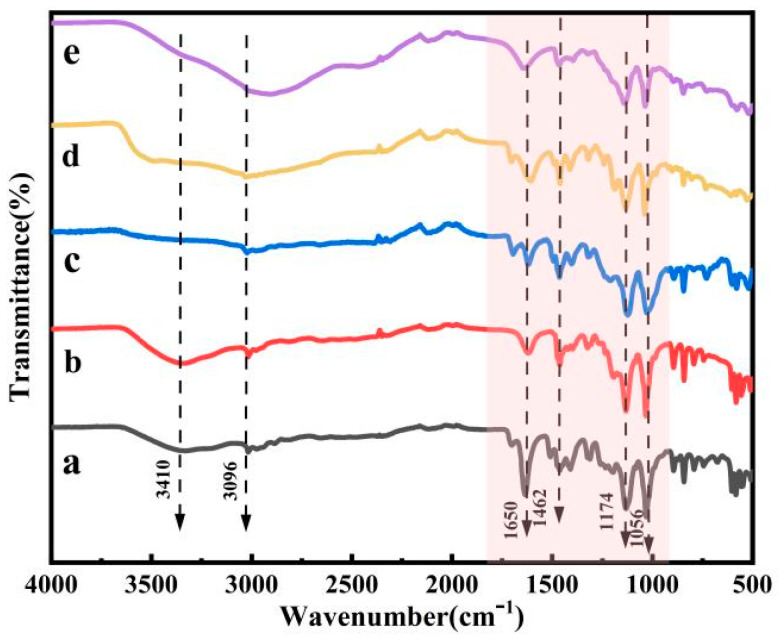
FT-IR spectra of five acidic metal-based functional ionic liquid catalysts. ((a) [DA-2PS][ZnCl_3_]_2_; (b) [DA-2PS][MnCl_3_]_2_; (c) [DA-2PS][FeCl_4_]_2_; (d) [DA-2PS][AlCl_4_]_2_; (e) [DA-2PS][SnCl_5_]_2_).

As demonstrated in Figure 3, the absorption peak at 3410 cm^−1^ corresponds to the O-H stretching vibration peak, while the characteristic peak at 3096 cm^−1^ represents the antisymmetric stretching vibration peak of C-H. Additionally, the absorption peak at 1462 cm^−1^ corresponds to the deformation vibration of the C-H bond. The absorption peak at 1174 cm^−1^ can be attributed to the S=O stretching vibration peak, and the absorption peak at 1056 cm^−1^ corresponds to the C-N stretching vibration peak. The absorption peak at 1650 cm^−1^ is due to the presence of water in the preparation of the ionic liquid. Therefore, it can be determined from the above preliminary analysis of FT-IR spectra that the preparation of acidic metal-based functional ionic liquid catalysts was successful.

### 3.2. Molecular Structure of Acidic Metal-Based Functional Ionic Liquid Catalysts

A ^1^H NMR spectrometer was used to examine the molecular structure of the acidic metal-based functional ionic liquid catalyst (taking the catalyst [DA-2PS][SnCl_5_]_2_ as an example).

**Figure 4:**
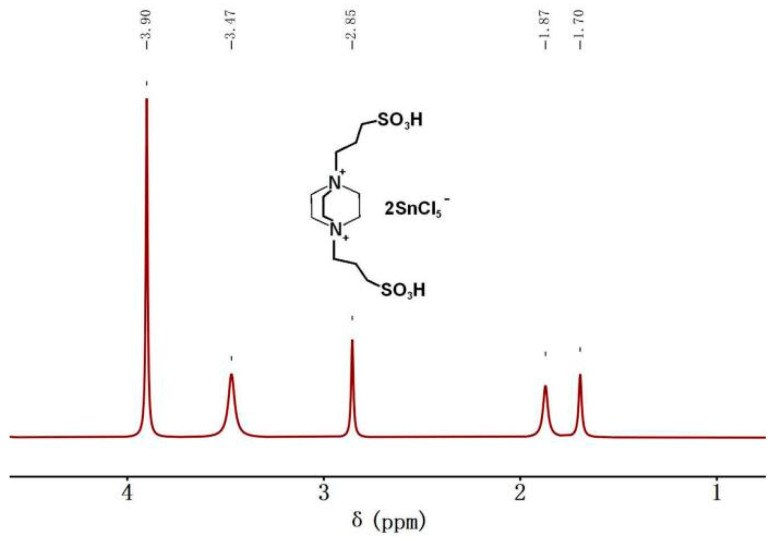
^1^H NMR spectrum (400 MHz, DMSO, 25 °C) of [DA-2PS][SnCl_5_]_2_.

According to Figure 4, it can be observed that the hydrogen atom at a chemical shift of δ = 3.90 ppm corresponds to the hydrogen atom on triethylenediamine. Similarly, the hydrogen atom at δ = 3.47 ppm corresponds to the hydrogen atom in the methylene group adjacent to -SO_3_^−^ on the chain. The hydrogen atom at δ = 2.85 ppm corresponds to the hydrogen atom in the intermediate methylene group on the chain. Moreover, the hydrogen atoms at δ = 1.87 ppm are associated with the methylene group close to N on the chain. The chemical shift at δ = 1.70 ppm was the peak of active hydrogen in the sulfonic acid group. It can be ultimately confirmed that acidic metal-based functional ionic liquid catalysts were prepared successfully. In addition, the analysis of the ^1^H NMR and FT-IR spectrum shows that the addition of SnCl_4_ does not change the structure of the ionic liquid, but only the ionic liquid cationic group complexes with the metal ions.

### 3.3. Crystallization Performance of Acidic Metal-Based Functional Ionic Liquid Catalysts

XRD was used to test acidic metal-based functional ionic liquid catalyst (taking the catalyst [DA-2PS][SnCl_5_]_2_ as an example).

**Figure 5:**
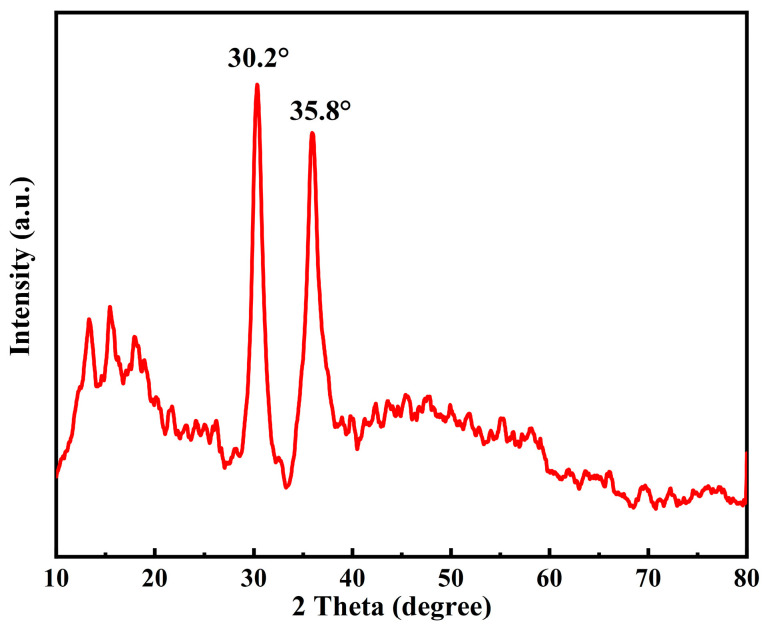
XRD diffraction spectra of [DA-2PS][SnCl_5_]_2_.

It can be seen from Figure 5 that [DA-2PS][SnCl_5_]_2_ showed the diffraction peaks of metal Sn at 2θ = 30.2° and 35.8°. There were no other crystal structures in [DA-2PS][SnCl_5_]_2_, so there were no other significant characteristic diffraction peaks. This also indicated that there was no structural failure in [DA-2PS][SnCl_5_]_2_. Therefore, the preparation of acidic metal-based functional ionic liquid catalysts was successful.

### 3.4. Thermal Performance of Acidic Metal-Based Functional Ionic Liquid Catalysts

Thermal performance tests were conducted on acidic metal-based functional ionic liquid catalyst (taking the catalyst [DA-2PS][SnCl_5_]_2_ as an example).

**Figure 6:**
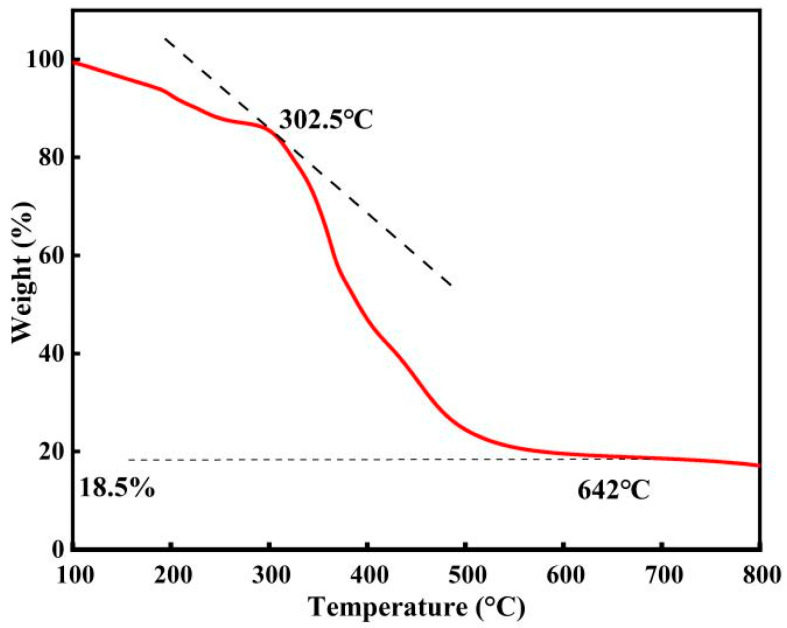
Thermogravimetric analysis of [DA-2PS][SnCl_5_]_2_. (N_2_, 10 °C/min).

As shown in Figure 6, [DA-2PS][SnCl_5_]_2_ experienced a slight mass loss due to residual water content when heated from room temperature to 302.5 °C, but this had negligible impact on the catalyst. With continued heating, the catalyst underwent further mass loss and decomposition, which was essentially completed at 642 °C and the residual catalyst was 18.5%. Overall, the acidic metal-based functional ionic liquid catalysts demonstrated excellent thermal stability, surpassing the maximum required reaction temperature for the esterification and polycondensation reaction. Hence, they fully satisfy the thermal stability criteria for catalyzing this reaction.

### 3.5. Pyridine Diacid Probe of Acidic Metal-Based Functional Ionic Liquid Catalysts

Pyridine was used as a probe molecule and the chemical structure of the substance produced by mixing pyridine and acidic metal-based functional ionic liquid catalysts was characterized using an FT-IR spectrometer (taking the catalyst [DA-2PS][SnCl_5_]_2_ as an example).

**Figure 7:**
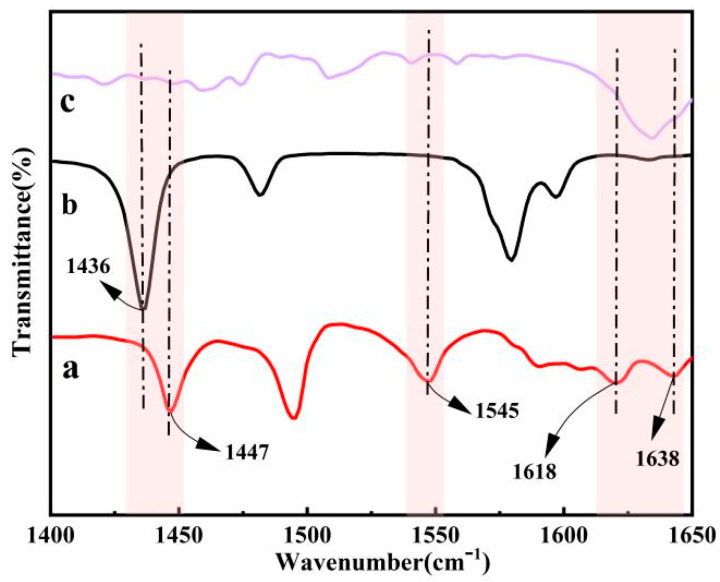
Analysis of pyridine probe curve of [DA-2PS][SnCl_5_]_2_. (a-pyridine/[DA-2PS][SnCl_5_]_2_; b-pure pyridine; c-[DA-2PS][SnCl_5_]_2_).

According to the analysis in Figure 7, the acidic metal-based functional ionic liquid catalyst [DA-2PS][SnCl_5_]_2_ reacts with pyridine as a probe. The electron pairs on the nitrogen atoms interact with both Brønsted acidic and Lewis acidic to produce a pyridine cation (PyH^+^) and coordination complex (Py-Lewis), respectively. The absorption peaks of PyH^+^ are observed at 1545 cm^−1^ and 1638 cm^−1^, while those of Py-Lewis are observed at 1447 cm^−1^ and 1618 cm^−1^. This indicates that the synthesized acidic metal-based functional ionic liquid catalysts exhibit dual Brønsted–Lewis acidity.

### 3.6. Effect of Catalyst Type on Catalytic Performance

Five acidic metal-based functional ionic liquid catalysts were prepared to catalyze the synthesis of PEF by direct esterification of FDCA with EG, and the catalytic performances were compared with those of catalyst-free, conventional catalysts and other ionic liquid catalysts. The catalyst dosage used was 0.15 mol% based on FDCA. The esterification reaction temperature was set at 190 °C, and the reaction time was determined by the temperature at the top of the kettle dropping below 80 °C after reaching the clarification point. The condensation reaction temperature was 240 °C, with a time of 5 h and a pressure of −0.1 MPa. The effects of different catalysts on FDCA conversion and the intrinsic viscosity of polyester PEF were investigated to evaluate their performance and the results are shown in Table 1.

Comparing the data in Table 1, it is evident that the catalytic performance of acidic metal-based functional ionic liquid catalysts surpasses that of ordinary catalysts and other ionic liquid catalysts with only one acidic group. This suggests that the catalysts’ effectiveness in promoting the reaction improves with an increase in the number of acidic sites. Additionally, the catalytic effect varies depending on the anions employed. When comparing [DA-2PS][Cl]_2_ in Table 1 with the five metal-based functional ionic liquid catalysts containing introduced metal groups, the inclusion of metal facilitates a synergistic catalytic effect between the Lewis acid metal and the Brønsted acidic sulfonic acid group. The catalytic effects of different Lewis acid species follow the order of SnCl_4_ > FeCl_3_ > ZnCl_2_ > MnCl_2_ > AlCl_3_. Among them, [DA-2PS][SnCl_5_]_2_ showed the best catalytic effect with an FDCA conversion of 97.8% and the intrinsic viscosity of the PEF product was 0.50 dL·g^−1^.

In summary, ionic liquid catalysts demonstrate excellent catalytic effects with an increase in acidic sites, and the introduction of Lewis acid metal in acidic metal-based functional ionic liquid catalysts enhances their catalytic performance through a synergistic effect.

### 3.7. Influence of Process Parameters on the Catalytic Process

#### 3.7.1. Effect of Catalyst Dosage on Catalytic Performance

**Figure 8:**
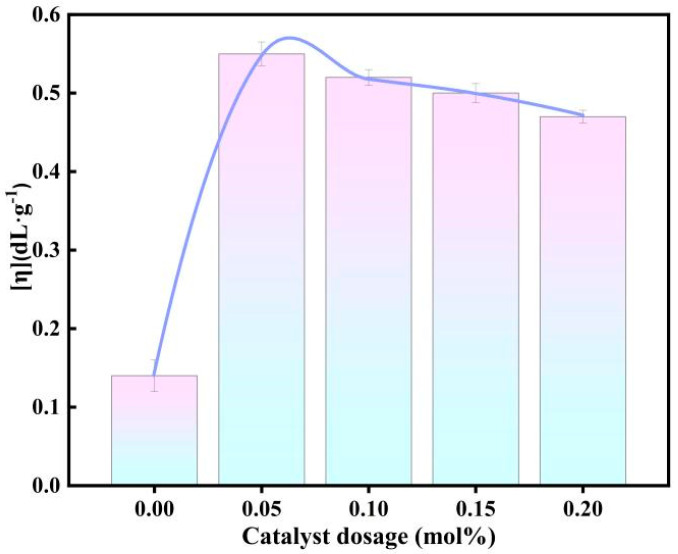
Effect of different catalyst dosages on intrinsic viscosity of PEF.

Since the catalytic performance of [DA-2PS][SnCl_5_]_2_ was the best under the same reaction conditions, the catalyst dosage was first investigated in a one-factorial manner. The specific reaction conditions and steps are shown in Section 2.3, the optimum catalyst dosage was explored by using the intrinsic viscosity of PEF as a measure and the intrinsic viscosity of the synthesized PEF with different catalyst dosages are shown in Figure 8.

The intrinsic viscosity of PEF increases and then decreases with the amount of catalyst. When the amount of catalyst is 0.05 mol% based on FDCA, the intrinsic viscosity of PEF reaches a maximum of 0.55 dL·g^−1^; when the amount of catalyst is further increased, the intrinsic viscosity of PEF begins to gradually decrease, which is due to the double effect of the amount of catalyst on the synthesis of PEF, which is a reversible reaction; the catalyst accelerates the positive reaction and catalyzes the side reaction at the same time. Therefore, the optimum amount of catalyst for [DA-2PS][SnCl_5_]_2_ catalysis is 0.05 mol% based on FDCA.

#### 3.7.2. Optimization of Optimal Process Parameters Using the Response Surface Methodology

In order to optimize the process parameters of this reaction and obtain more accurate data, we used the Response Surface Method (RSM) in the Box–Behnken model (BBD) in the Design-Expert software. The feeding ratio, esterification temperature, polycondensation temperature and polycondensation time of the reaction system for the direct esterification method of FDCA and EG in the preparation of polyester PEF were labeled as Factors A, B, C and D, respectively. The intrinsic viscosity of the polyester PEF was considered as the response value Y. Graphs were utilized to display the functional relationship among the factors, and to determine the optimal conditions of the experimental design. The results of the full factorial BBD design with three levels of the four factors and the intrinsic viscosity of the polyester PEF are presented in Table 2.

Statistical tests of the model were performed with analysis of variance (ANOVA) and the results at the coded variable level are shown in Table 3. The model F = 18.40, *p* < 0.0001, indicating that the quadratic regression model is highly significant. The linear relationship between the response value Y and the influencing factors A, B, C and D is evident, and the regression model can be used to predict the intrinsic viscosity of polyester PEF. Moreover, the correlation coefficient R^2^ of the equation is 0.9810, suggesting that this model can explain the majority of the data.

**Figure 9:**
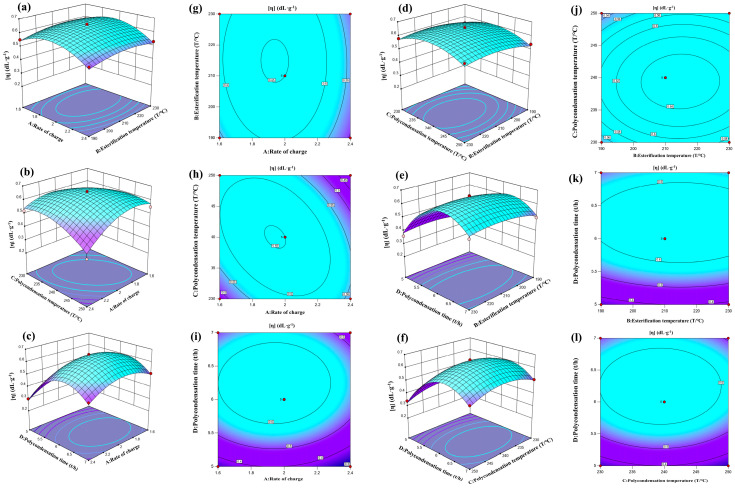
Diagram of RSM between the various factors. (**a**–**f**) 3D response surface plots and (**g**–**l**) Contour.

The influence of the four factors, feeding ratio (A), esterification temperature (B), polycondensation temperature (C) and polycondensation time (D), on the intrinsic viscosity of polyester was analyzed using a 3D response surface. The X-axis and Y-axis represented two factors, and six response curves and six contour results were obtained (Figure 9). For example, in Figure 9a,b, it can be observed that the intrinsic viscosity of polyester initially increases and then decreases with an increase in the feeding ratio when the esterification time is constant. Similarly, when the feeding ratio is fixed, the intrinsic viscosity shows a slight increase and then decreases with an increase in the esterification temperature. However, the overall trends are not significant. The curve corresponding to the feeding ratio is steeper than that of the esterification temperature, indicating that the feeding ratio has a greater impact on the intrinsic viscosity of polyester compared to the esterification temperature. The contour plot also suggests that the interaction between the two factors is not significant, which is consistent with the software results. Based on the provided graphs, the order of significance of the four factors on the intrinsic viscosity of polyester is as follows: polycondensation time (D) > feeding ratio (A) > esterification temperature (B) > polycondensation temperature (C).

The regression model was analyzed using the response surface optimization method, and the optimal reaction conditions were determined as follows: a feeding ratio of 1.962:1, an esterification temperature of 219.864 °C, a polycondensation temperature of 240.038 °C and a polycondensation time of 6.3 h. The predicted intrinsic viscosity of the polyester PEF was 0.67 dL·g^−1^. To validate the reliability of these predicted values, three sets of parallel experiments were conducted under the optimized conditions (as shown in Table 4). The experimental results showed that the polyester PEF intrinsic viscosity was 0.68 dL·g^−1^, which closely matched the predicted value. This indicates that the response surface analysis is accurate and reliable for optimizing process parameters, and it can provide valuable insights for the design of subsequent processes.

### 3.8. Characterization of Polyester PEF

#### 3.8.1. FT-IR Structure Characterization of PEF

The polymerization product PEF was characterized by FT-IR structure.

**Figure 10:**
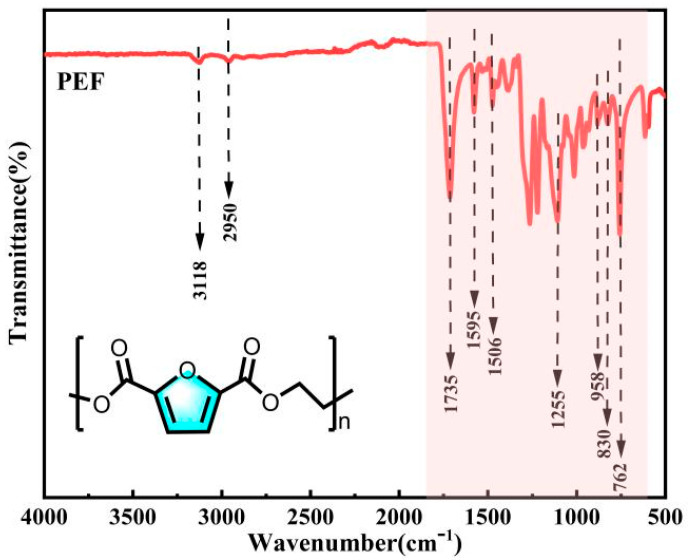
FT-IR spectra of PEF.

From the analysis of Figure 10, it can be observed that the telescopic vibration peaks of the furan ring C-H occur at 3118 cm^−1^, -CH_2_ at 2950 cm^−1^, C=O at 1735 cm^−1^, and furan ring C=C at 1595 cm^−1^ and 1506 cm^−1^. The peak at 1255 cm^−1^ corresponds to the C-O-C stretching vibration, while the peaks at 958 cm^−1^, 830 cm^−1^ and 762 cm^−1^ represent the bending vibrations of the furan ring. In conclusion, the infrared spectra of the products were analyzed and compared with the standard PEF infrared spectra in the database. The peak shapes and positions of the characteristic peaks in the products were consistent with the standard PEF spectra, confirming that the polymer products were PEF.

#### 3.8.2. Hydrogen-Nuclear Magnetic Structure Characterization of PEF

PEF weas characterized by ^1^H NMR structure.

**Figure 11:**
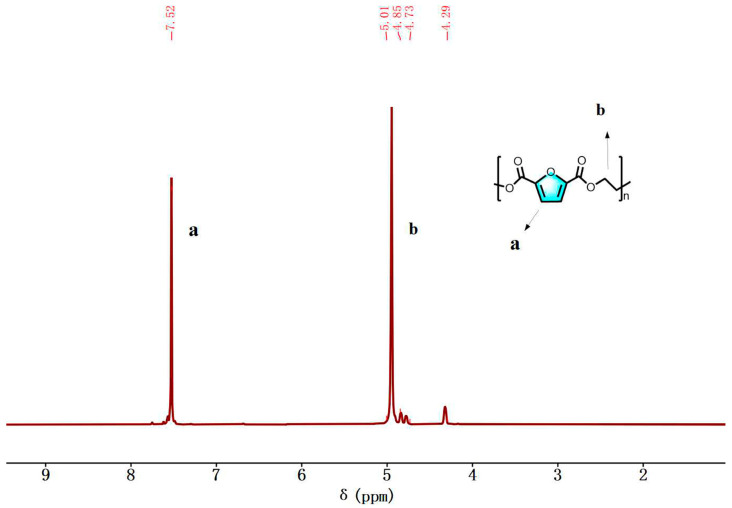
^1^H NMR spectra (400 MHz, TFA, 25 °C) of PEF.

The analysis of Figure 11 reveals several proton peaks in the ^1^H NMR spectrum of PEF. The proton peak on the furan ring of PEF is observed at δ = 7.52 ppm, while the proton peaks on -OCH_2_- and -CH_2_- in the chain segment are observed at δ = 4.95 ppm.

Additionally, extra chemical shifts were also observed at 4.78 ppm and 4.29 ppm. These signals are attributed to methylene protons in the DEGF unit, which are by-products of etherification of EG or hydroxyethyl ester end groups. The number of atoms calculated by integration is consistent with the molecular design. It can be ultimately confirmed that PEF was prepared successfully.

#### 3.8.3. Thermal Stability Analysis of PEF

TGA tests were carried out for PEF.

**Figure 12:**
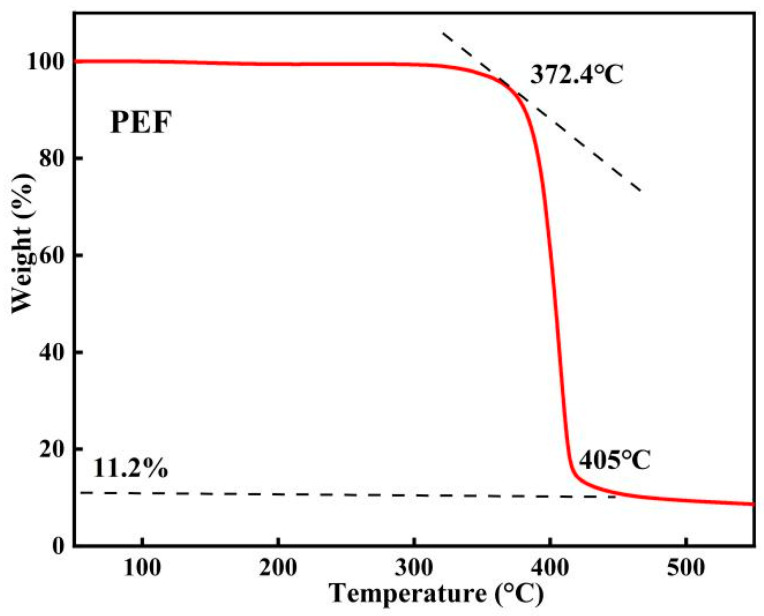
TGA curve of PEF. (N_2_, 10 °C/min).

From the TG curve in Figure 12, it is evident that the thermal weight loss process of polymer PEF can be categorized into three stages. In the first stage (from room temperature to 372.4 °C), PEF experienced a small mass change. In the second stage (from 372.4 °C to 405 °C), PEF underwent a significant mass loss and began to decompose rapidly. In the third stage (from 405 °C to 520 °C), there was no longer any significant change in the mass of PEF. In this case, the residual mass was about 6.7% of the total mass. In summary, PEF demonstrates high thermal stability and can be processed using standard thermal processing equipment.

#### 3.8.4. Differential Scanning Calorimetry Analysis of PEF

DSC tests were carried out for PEF.

**Figure 13:**
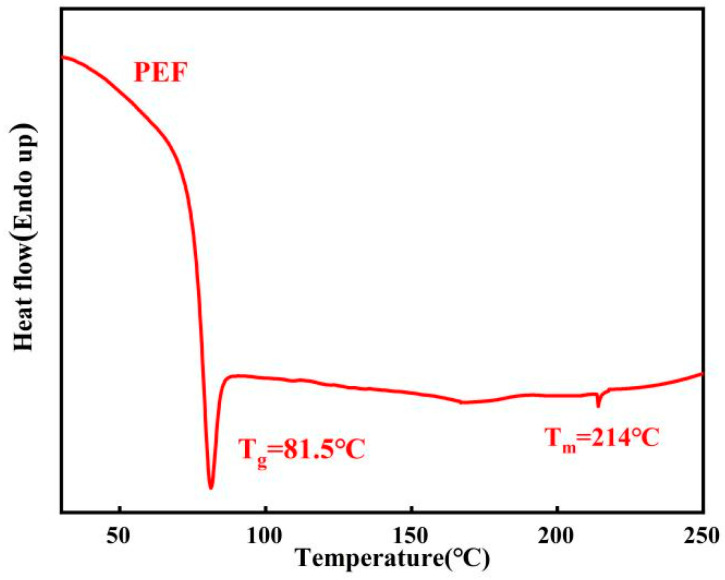
DSC curves of PEF (second heating scan at 10 °C/min).

Based on the analysis of Figure 13, it is evident that the synthetic PEF has a T_g_ (glass transition temperature) of 81.5 °C and a melting point of 214 °C. PEF exhibits a relatively higher T_g_ and lower T_m_. This characteristic enables PEF products to have a higher operating temperature while requiring a lower processing temperature, thereby expanding its potential application areas and reducing processing costs.

## 4. Conclusions

In this paper, we successfully prepared five acidic metal-based functional ionic liquid catalysts, namely [DA-2PS][ZnCl_3_]_2_, [DA-2PS][MnCl_3_]_2_, [DA-2PS][FeCl_4_]_2_, [DA-2PS][SnCl_5_]_2_ and [DA-2PS][AlCl_4_]_2_. The structural correctness of the catalysts was confirmed by FT-IR, ^1^H NMR and XRD. TGA revealed excellent thermal stability performance. The catalyst exhibited dual acidity as determined by pyridine infrared probe. The catalytic performance of the catalysts was investigated, and [DA-2PS][SnCl_5_]_2_ was found to be the most effective catalyst under the same conditions. Subsequently, we analyzed the optimal conditions using response surface analysis; the results showed that optimal process parameters included a catalyst dosage of 0.05 mol% based on FDCA, a feed ratio of EG:FDCA = 1.96:1, an esterification temperature of 219.86 °C, a polycondensation temperature of 240.04 °C and a polycondensation time of 6.3 h. We conducted experiments under these optimal process parameters and obtained a polyester PEF with an intrinsic viscosity of 0.68 dL·g^−1^, which was in agreement with the results predicted by the response surface. Finally, the PEF synthesized under optimal process conditions was characterized. The FT-IR and ^1^H NMR characterization results confirmed its correct structure. The decomposition temperature was determined by the TGA, demonstrating its excellent thermal stability property. Additionally, the DSC curve analysis revealed that PEF has a higher T_g_ and lower T_m_. This study provides new ideas and data support for the field of green and efficient catalysis of bio-based polyester PEF.

## Figures and Tables

**Table 1:** Effects of different catalyzers on catalytic performance.

No.	Catalyst	Conversion Rates/%	[η]/dL·g^−1^
1	—	92.2	0.14
2	DBU	93.4	0.38
3	(CH_3_COO)_2_Mn	94.6	0.41
4	[HO_3_S-(CH_2_)_3_-mim]-Cl	93.1	0.35
5	[HO_3_S-(CH_2_)_3_-mim]-SnCl_5_	94.1	0.39
6	[DA-2PS][Cl]_2_	94.8	0.40
7	[DA-2PS][ZnCl_3_]_2_	95.9	0.44
8	[DA-2PS][MnCl_3_]_2_	95.5	0.42
9	[DA-2PS][FeCl_4_]_2_	97.1	0.48
10	[DA-2PS][SnCl_5_]_2_	97.8	0.50
11	[DA-2PS][AlCl_4_]_2_	95.5	0.41

**Table 2:** Response surface test design and results.

Run	Factor				[η]/dL·g^−1^
	Raw Material Ratio (A) N (EG)/n (FDCA)	Esterification Temperature (B) (°C)	Polycondensation Temperature (C) (°C)	Polycondensation Time (D) (h)	
1	1.6	190	240	6	0.55
2	2.4	190	240	6	0.51
3	1.6	230	240	6	0.58
4	2.4	230	240	6	0.53
5	2	210	230	5	0.35
6	2	210	250	5	0.34
7	2	210	230	7	0.51
8	2	210	250	7	0.50
9	1.6	210	240	5	0.35
10	2.4	210	240	5	0.30
11	1.6	210	240	7	0.51
12	2.4	210	240	7	0.47
13	2	190	230	6	0.55
14	2	230	230	6	0.58
15	2	190	250	6	0.54
16	2	230	250	6	0.58
17	1.6	210	230	6	0.47
18	2.4	210	230	6	0.52
19	1.6	210	250	6	0.55
20	2.4	210	250	6	0.37
21	2	190	240	5	0.33
22	2	230	240	5	0.36
23	2	190	240	7	0.50
24	2	230	240	7	0.53
25	2	210	240	6	0.64
26	2	210	240	6	0.65
27	2	210	240	6	0.66
28	2	210	240	6	0.65
29	2	210	240	6	0.65

**Table 3:** Results of the analysis of variance.

Source	Sum of Squares	Degrees of Freedom	Mean Square	F-Value	*p*-Value	Significance
Model	0.3146	14	0.0225	51.65	<0.0001	significant
A	0.008	1	0.008	18.40	0.0007	
B	0.0027	1	0.0027	6.21	0.0259	
C	0.0008	1	0.0008	1.92	0.1881	
D	0.0817	1	0.0817	187.71	<0.0001	
AB	0	1	0	0.0575	0.814	
AC	0.0132	1	0.0132	30.39	<0.0001	
AD	0	1	0	0.0575	0.8140	
BC	0	1	0	0.0575	0.8140	
BD	0	1	0	0	1	
CD	0	1	0	0	1	
A^2^	0.0473	1	0.0473	108.76	<0.0001	
B^2^	0.0065	1	0.0065	14.95	0.0017	
C^2^	0.0288	1	0.0288	66.26	<0.0001	
D^2^	0.1829	1	0.1829	420.33	<0.0001	
Residual	0.0061	14	0.0004			
Lack of Fit	0.0059	10	0.0006	11.78	0.0148	significant
Pure Error	0.0002	4	0.0001			
Cor Total	0.3207	28				
	R^2^ = 0.9810			Adjusted R^2^ = 0.9620		

**Table 4:** Best process conditions and verification results.

Raw Material Ration (EG)/n (FDCA)	Esterification Temperature (°C)	Polycondensation Temperature (°C)	Polycondensation Time (h)	Predicted [η]/dL·g^−1^	Actual [η]/dL·g^−1^
1.962:1	219.864	240.038	6.300	0.67	0.66
					0.70
					0.68
average					0.68

## Data Availability

Data are contained within the article.

## References

[1] Mendieta C.M., González G., Vallejos M.E., Area M.C. (2022). Bio-polyethylene furanoate (Bio-PEF) from lignocellulosic biomass adapted to the circular bioeconomy. BioResources.

[2] Sousa A.F., Coelho J.F.J., Silvestre A.J.D. (2016). Renewable-based poly ((ether) ester)s from 2,5-furandicarboxylic acid. Polymer.

[3] Post C., Maniar D., Voet V.S.D., Folkersma R., Loos K. (2023). Biobased 2,5-Bis (hydroxymethyl) furan as a Versatile Building Block for Sustainable Polymeric Materials. ACS Omega.

[4] Burgess S.K., Kriegel R.M., Koros W.J. (2015). Carbon dioxide sorption and transport in amorphous poly (ethylene furanoate). Macromolecules.

[5] Van Berkel J., Guigo N., Kolstad J., Sipos L., Wang B., Dam M., Sbirrazzuoli N. (2015). Isothermal crystallization kinetics of poly (ethylene 2,5-furandicarboxylate). Macromol. Mater. Eng..

[6] Wu J.P., Xie H.Z., Wu L.B., Li B.G., Dubois P. (2017). DBU-catalyzed biobased poly(ethylene 2,5-furandicarboxylate) polyester with rapid melt crystallization: Synthesis, crystallization kinetics and melting behavior. RSC Adv..

[7] Knoop R.J.I., Vogelzang W., Haveren J.V., Es D. (2013). High molecular weight poly(ethylene-2,5-furanoate); Critical aspects in synthesis and mechanical property determination. J. Polym. Sci. Polym. Chem..

[8] Jiang M., Liu Q., Zhang Q., Ye C., Zhou G.Y. (2012). A series of furan-aromatic polyesters synthesized via direct esterification method based on renewable resources. J. Polym. Sci. Polym. Chem..

[9] Hill J.W., Carothers W.H. (1933). Studies of Polymerization and Ring Formation. XX. Many-Membered Cyclic Esters. J. Am. Chem. Soc..

[10] Morales-Huerta J.C., deIlarduya A.M., Muñoz-Guerra S. (2016). Poly (alkylene 2,5-furandicarboxylate) s (PEF and PBF) by ring opening polymerization. Polymer.

[11] Moore J.A., Kelly J.E. (1978). Polyesters derived from furan and tetrahydrofuran nuclei. Macromolecules.

[12] Gandini A., Coelho D., Gomes M., Reis B., Silvestre A. (2009). Materials from renewable resources based on furan monomers and furan chemistry: Work in progress. J. Mater. Chem..

[13] Gomes M., Gandini A., Silvestre A.J., Reis B. (2011). Synthesis and characterization of poly (2,5-furan dicarboxylate) s based on a variety of diols. J. Polym. Sci. Pol. Chem..

[14] Jiang Y., Woortman A.J., Alberda van Ekenstein G.O., Petrovic D.M., Loos K. (2014). Enzymatic synthesis of biobased polyesters using 2,5-bis (hydroxymethyl) furan as the building block. Biomacromolecules.

[15] Hong S., Min K.D., Nam B.U., Park O.O. (2016). High molecular weight bio furan-based co-polyesters for food packaging applications: Synthesis, characterization and solid-state polymerization. Green Chem..

[16] Terzopoulou Z., Karakatsianopoulou E., Kasmi N., Majdoub M., Papageorgiou G.Z., Bikiaris D.N. (2017). Effect of catalyst type on recyclability and decomposition mechanism of poly (ethylene furanoate) biobased polyester. J. Anal. Appl. Pyrolysis..

[17] Gruter G.J.M., Sipos L., Adrianus Dam M. (2012). Accelerating research into bio-based FDCA-polyesters by using small scale parallel film reactors. Comb. Chem. High Throughput Screen..

[18] Martino L., Guigo N., van Berkel J.G., Sbirrazzuoli N. (2017). Influence of organically modified montmorillonite and sepiolite clays on the physical properties of bio-based poly (ethylene 2,5-furandicarboxylate). Compos. Part B Eng..

[19] Joshi A.S., Alipourasiabi N., Kim Y.W., Coleman M.R., Lawrence J.G. (2018). Role of enhanced solubility in esterification of 2,5-furandicarboxylic acid with ethylene glycol at reduced temperatures: Energy efficient synthesis of poly (ethylene 2, 5-furandicarboxylate). React. Chem. Eng..

[20] Gopalakrishnan P., Narayan-Sarathy S., Ghosh T., Mahajan K., Belgacem M.N. (2014). Synthesis and characterization of bio-based furanic polyesters. J. Polym. Res..

[21] Gubbels E., Jasinska-Walc L., Noordover B.A., Koning C.E. (2013). Linear and branched polyester resins based on dimethyl-2, 5-furandicarboxylate for coating applications. Eur. Polym. J..

[22] Wang J., Liu X., Zhu J., Jiang Y. (2017). Copolyesters based on 2, 5-furandicarboxylic acid (FDCA): Effect of 2, 2, 4, 4-tetramethyl-1, 3-cyclobutanediol units on their properties. Polymers.

[23] Wang J., Liu X., Zhang Y., Liu F., Zhu J. (2016). Modification of poly (ethylene 2, 5-furandicarboxylate) with 1, 4-cyclohexanedimethylene: Influence of composition on mechanical and barrier properties. Polymer.

[24] Wu J., Eduard P., Thiyagarajan S., Noordover B.A., van Es D.S., Koning C.E. (2015). Semi-Aromatic Polyesters Based on a Carbohydrate-Derived Rigid Diol for Engineering Plastics. ChemSusChem.

[25] Miao L., Song Z., Zhu D., Li L., Gan L., Liu M. (2021). Ionic liquids for supercapacitive energy storage: A mini-review. Energy Fuels..

[26] Yan S., Han F., Hou Q., Zhang S., Ai S. (2019). Recent advances in ionic liquid-mediated SO_2_ capture. Ind. Eng. Chem. Res..

[27] Li J., Peng X., Luo M., Zhao C.J., Gu C.B., Zu Y.G., Fu Y.J. (2014). Biodiesel production from Camptotheca acuminata seed oil catalyzed by novel Brönsted–Lewis acidic ionic liquid. Appl. Energy.

[28] Gupta S., Singh P.K., Bhattacharya B. (2018). Low-viscosity ionic liquid-doped solid polymer electrolytes: Electrical, dielectric, and ion transport studies. High Perform. Polym..

[29] Zhao X.D., Guo L.Y., Xu T.J., Zheng R.R., Wang H.Y. (2022). Preparation of Keggin-type monosubstituted polyoxometalate ionic liquid catalysts and their application in catalyzing the coupling reaction of ethylene carbonate and dimethyl succinate to synthesize poly (ethylene succinate). N. J. Chem..

[30] Ma C., Xu F., Cheng W., Tan X., Su Q., Zhang S.J. (2018). Tailoring molecular weight of bioderived polycarbonates via bifunctional ionic liquids catalysts under metal-free conditions. ACS Sustain. Chem. Eng..

[31] Zhang Z.C., Xu F., He H.Y., Ding W., Fang W.J., Sun W., Zhang S. (2019). Synthesis of high-molecular weight isosorbide-based polycarbonates through efficient activation of endo-hydroxyl groups by an ionic liquid. Green Chem..

[32] Peng Q.H., Wei L., Zhang X.Y., Wu Y., Mahmood K., Liu Z.P., Zhang L.S. (2021). Direct polycondensation of l-lactic acid in hydrophobic bis (trifluoromethanesulfonyl) imide-anionic ionic liquids: A kinetic study. Eur. Polym. J..

[33] Zhao X.D., Guo L.Y., Xu T.J., Wang H.Y., Zheng R.R., Jiang Z.Z. (2022). Preparation of biacidic tin-based ionic liquid catalysts and their application in catalyzing coupling reaction between ethylene carbonate and dimethyl succinate to synthesize poly (ethylene succinate). N. J. Chem..

[34] Qu X.L., Jiang M., Wang B., Deng J., Wang R., Zhang Q., Tang J. (2019). A Brønsted Acidic Ionic Liquid as an Efficient and Selective Catalyst System for Bioderived High Molecular Weight Poly (ethylene 2, 5-furandicarboxylate). ChemSusChem.

[35] (1989). Determination of Limiting Viscosity Number of Polyacrylamide.

[36] Xie H.Z., Wu L.B., Li B.G., Dubois P. (2018). Biobased poly(ethylene-co-hexamethylene 2,5- furandicarboxylate) (PEHF) copolyesters with superior tensile properties. Ind. Eng. Chem. Res..

